# A novel approach to assess acid diversion efficiency in horizontal wells

**DOI:** 10.1038/s41598-024-84671-y

**Published:** 2025-01-07

**Authors:** Abdulameer Almalichy, Murtada Saleh Aljawad, Zoltan Turzo, Ahmed Al-Yaseri

**Affiliations:** 1 Institute of Mining and Energy, Faculty of Earth and Environmental Sciences and Engineering, University of Miskolc, Miskolc-Egyetemvaros, 3515 Hungary; 2 Department of Petroleum Engineering, King Fahd University of Petroleum & Minerals, Dhahran, 31261 Saudi Arabia; 3 Center for Integrative Petroleum Research, King Fahd University of Petroleum & Minerals, Dhahran, 31261 Saudi Arabia

**Keywords:** Matrix acidizing, Diversion, Viscoelastic surfactant (VES), Carbonate formation, Wormhole, Energy, Chemical engineering, Crude oil

## Abstract

Using an acid to stimulate a heterogeneous carbonate reservoir during matrix acidizing may lead to over-treating the high permeability zones, leaving low permeability zones untreated. This is particularly exacerbated in long horizontal sections, necessitating the use of acid diverters for effective acid distribution across the formation. In previous studies, conventional core flooding systems were utilized where single inlet and outlet lines were used or, at best, two outlet lines for dual-core flooding. This paper proposes a new method for simulating matrix acidizing in horizontal wells by introducing five injection points and two outlet lines. The injection points are perpendicular to the core samples to simulate multiple perforations in a horizontal well while the outlet lines are parallel. Four experiments were conducted in this study using Indiana limestone cores that were 1.5 inches in diameter. For the first three tests, the length of the core was 12 inches, and the cores’ average permeabilities were 16 mD. For the fourth one, two 6-inch length cores with different average permeability (10 and 50 mD) were employed. Hydrochloric acid was used in the first experiment, while hydrochloric acid with viscoelastic surfactant (VES) was used in subsequent experiments. To the best of our knowledge, this is the first study to introduce a multi-point injection system with enhanced coverage and distribution, resulting in a more precise representation of acidizing a horizontal well.

## Introduction

Matrix acidizing in a carbonate reservoir is the process of injecting acid pressure below the fracture pressure to remove formation damage and create new flow channels called wormholes^1,2^. In rocks with varying properties, a significant difference in permeability may significantly decrease the effectiveness of stimulation treatments since the acid will predominantly flow into the zones with higher permeability. This phenomenon, similar to gas fingering behavior observed during CO2 flooding^3^, highligts the need for effective flow management strategies to ensure uniform treatment. Inadequate design will result in unequal treatment of target areas and an unsuccessful treatment with acid^4^. Further more, rock hetrogenity including spatial distribution and mineral composition strongly effect the reactive flow dynamics of acid^5^. This phenomenon will be worse in a thick or horizontal reservoir. Consequently, the industry has extensively adopted mechanical and chemical diverters, selecting the most effective approach to mitigating this effect for each lithology^6^.

Mechanical diverters are essential in matrix acidizing operations since they selectively redirect the acid flow to areas that have not been treated. This improves the effectiveness and consistency of the stimulation process. These diverters, including coil tubing, ball sealers, bridge plugs, and packers, are used to establish temporary barriers in the wellbore^7–10^. Bridge plugs and packers are only suitable for cased holes, and their utilization is associated with high costs^11^. Ball sealers are small spherical devices injected into well-treating fluids during oil and gas operations. They have an essential purpose in improving well productivity and optimizing stimulation treatments. Their primary function is establishing contact and closing perforations in the wellbore, which serve as channels for fluid flow from the reservoir into the well. Ball sealers have numerous benefits that have fundamentally transformed well completions. First and foremost, they offer a financially efficient option to conventional packers, resulting in substantial cost reductions in multistage fracture procedures while attaining desired outcomes. In addition, operators can target specific perforations for treatment using ball sealers. This ensures that only the targeted zones receive stimulation, enhancing the overall performance of the reservoir^12^. Furthermore, ball sealers are specifically engineered to effectively redirect treatment fluids toward the accepting perforations, guaranteeing ideal contact and sealing^7^.

Coiled tubing provides high speed, economic advantages, and effective methods for well-intervention procedures. Coil tubing (CT) can install mechanical diverters and transport acid into the wellbore, offering enhanced flexibility and efficiency in executing treatments. It has a compact design, resulting in reduced time for rigging up/down the equipment. Employing CT also minimizes well-site preparation, production downtime, environmental impacts, and staff requirements, thus reducing costs^13^. Moreover, when combined with foam or temporarily crosslinked gelled acid (TCGA) diversion, placing CT leads to enhanced zone coverage and a more consistent injection/production profile^10^.

In addition to mechanical diversion, chemical diversion technology has gradually developed as an alternative approach. Chemical diversion technology involves the addition of a diversion agent to the acid, including a chemical particle, gel (polymer-based gelled/in-situ gelled acid), surfactant (emulsified diversion acid, foam diversion acid, and self-diversion acid (SDA))^12,14^.

Polymer-based in-situ gelled acid, in which the solution consists of hydrochloric acid (HCl), a polymer, a crosslinker, and a breaker, has been utilized for acid diversion. In these systems, the viscosity of the acid increases upon contact with the carbonate rock, enabling the acid to divert more efficiently into less permeable zones^4,15^. In contrast, linear gel acid has been utilized for diversion, and consists of HCl and a polymer, without a crosslinker and a breaker^16–19^. However, polymer-based systems present challenges such as potential formation damage due to unbroken polymer residues and complications from iron-based crosslinkers, which can cause scaling and sludge formation^20^. Recent developments utilized inorganic nanoparticles as a gelling agent to address these issues. One nanoparticle-based in-situ gelled acid system can operate effectively at 5–20% acid concentrations and temperatures up to 300 °F^21^. Another notable advancement involves using non-iron-based metallic crosslinkers designed for high-temperature applications, which maintain stable gelation at temperatures up to 275 °F. These crosslinkers are effective in low pH environments (1.5-3) and break down as the pH rises, thus preventing long-term formation damage^20^.

Foam is also utilized as a diverter agent^22–26^. Foam restricts the movement of gas by effectively capturing a significant amount of gas in place. This trapping effect can retain 80–99% of the gas, even when the foam flows under high-pressure conditions. This capability makes foam an effective method for diverting acid away from high-permeability layers towards low-permeability or damaged zones, thus optimizing the acid distribution within the reservoir. In high-permeability layers, foam decreases liquid flow and the relative permeability of the liquid, thereby reducing acid flow into foam-saturated layers. However, foam exhibits less stability in low-permeability or damaged layers, allowing acid to penetrate without zonal isolation^27^. Foam can also be combined with VES to get a foam-plugging and viscosity effect of VES, offering dual benefits in acid diversion^28^.

Foam diversion methods often suffer from stability issues and inconsistent performance, especially surface-generated foam, which faces challenges during the pumping. Recent advancements have developed in-situ foam generation using thermochemical fluids, which create foam directly within the reservoir to address these problems. This approach improves foam stability, simplifies operations, and enhances acid diversion. These studies show that in-situ foam generation can increase well injectivity by up to 18-fold and reduce treatment costs by 65% compared to a viscoelastic diverting system, making it particularly effective in long horizontal wells^29,30^.

Self-diverting acid is utilized in several fields due to its minimal impact on the formation and excellent ability to divert the acid from a high permeability interval into a low one^31^. The self-diverting acid system primarily comprises acid and viscoelastic surfactants. The injection of VES as a diverter into the formation matrix enhances flow obstruction and lowers the effective permeability to water- and acid-based fluids. Consequently, more oil flows out than water, resulting in reduced water cut during the well’s production^32^. The viscoelastic acid system can achieve self-viscosification through self-consumption. The reaction of rock minerals with acid elevates the pH level and raises the calcium ions (Ca^2+^) concentration in the system. Surfactant molecules transform from spherical to rod-shaped. When neutralized with acid, a negative charge on the amphoteric surfactant molecule leads to a strong electrostatic attraction between the surfactant molecules and the Ca^2+^ ions, which carry two positive charges. This attraction causes the spherical micelles to become entangled and form wormlike micelles, resulting in high viscosity^33–35^.

Advancements in VES technology have focused on enhancing thermal stability and additive compatibility. Ibrahim et al. [34] tested betaine-based, sultaine-based, and aminoxide-based VES formulations designed for high-temperature carbonate reservoirs. These formulations maintained high viscosity at temperatures up to 300 °F and showed compatibility with corrosion inhibitors, preventing phase separation and ensuring consistent acid diversion.​ Another study presents a novel approach to improving acid diversion in carbonate rocks using thermochemical fluids. This method generates nitrogen gas in situ, which helps divert the injected acid into low-permeability formations more effectively. Experimental and numerical studies demonstrated that thermochemical fluids can create wormholes in high and low-permeability rocks, achieving a more uniform acid distribution^6^.

In dual-core flooding experiments, researchers utilize a setup consisting of two core holders connected in parallel. The core holders are loaded with two samples with varying permeabilities, one with high permeability and the other with low permeability, representing the heterogeneous reservoir. Through dual core flooding systems, researchers can assess the performance of different diversion materials, assessing their effectiveness in redirecting acid flow to less permeable zones and enhancing the acid distribution across heterogeneous reservoirs^6,28^.

In previous research, the acid injection and outlet points were uniaxial, and only a single injection point was used^28,32,33,36–41^. This study presents a novel acid stimulation system design with five injection spots representing a horizontal well’s perforated points. In addition, the injection points are positioned perpendicular to the core samples, which more accurately reflects the generated wormholes in actual horizontal wells. The new system can also evaluate the acid diversion efficiency in heterogeneous carbonate samples. The findings of this study have significant practical implications for field applications, particularly in the stimulation of horizontal wells. The research highlights how different acid systems influence the development and propagation of wormholes within the rock.

## Materials, setup, and experimental procedure

### Core samples

Indiana limestone core samples were utilized in this study. After obtaining the core samples from the source, they were cleaned, dried, and cut to the desired lengths of 6 and 12 inches. The porosity of the samples was measured (see Table 1). The mineralogy of the samples was determined by conducting XRD, which showed 100% calcite content.

### Chemicals

Deionized water is used for saturation and pre- and post-flooding. The acid systems used in the experiments are summarized in Table 1. The acid system used in experiments 2, 3 and 4 was prepared by adding 10 wt% of calcium chloride (CaCl_2_) to a solution consisting of 15 wt% of hydrochloric acid (HCl) plus 1 wt% corrosion inhibitor (CI) and mixing it by using an overhead stirrer made of corrosion-resistant titanium. Then, 6 wt % of viscoelastic surfactant (VES) was added gradually to the solution. The acid system used in the first experiment consisted of only 15 wt% HCl mixed with 1 wt% CI. To investigate the flow behavior of the viscoelastic surfactant, the viscosity was measured across a range of shear rates, from 100 to 1000 s⁻¹. The results show a non-linear relationship between shear rate and viscosity. As the shear rate increased, the viscosity of the viscoelastic surfactant decreased, with values of 153 mPa·s at 100 s⁻¹, 113 mPa·s at 250 s⁻¹, and continuing to decline to 28 mPa·s at 1000 s⁻¹.

### Experimental setup

#### Core flooding

**Fig. 1:**
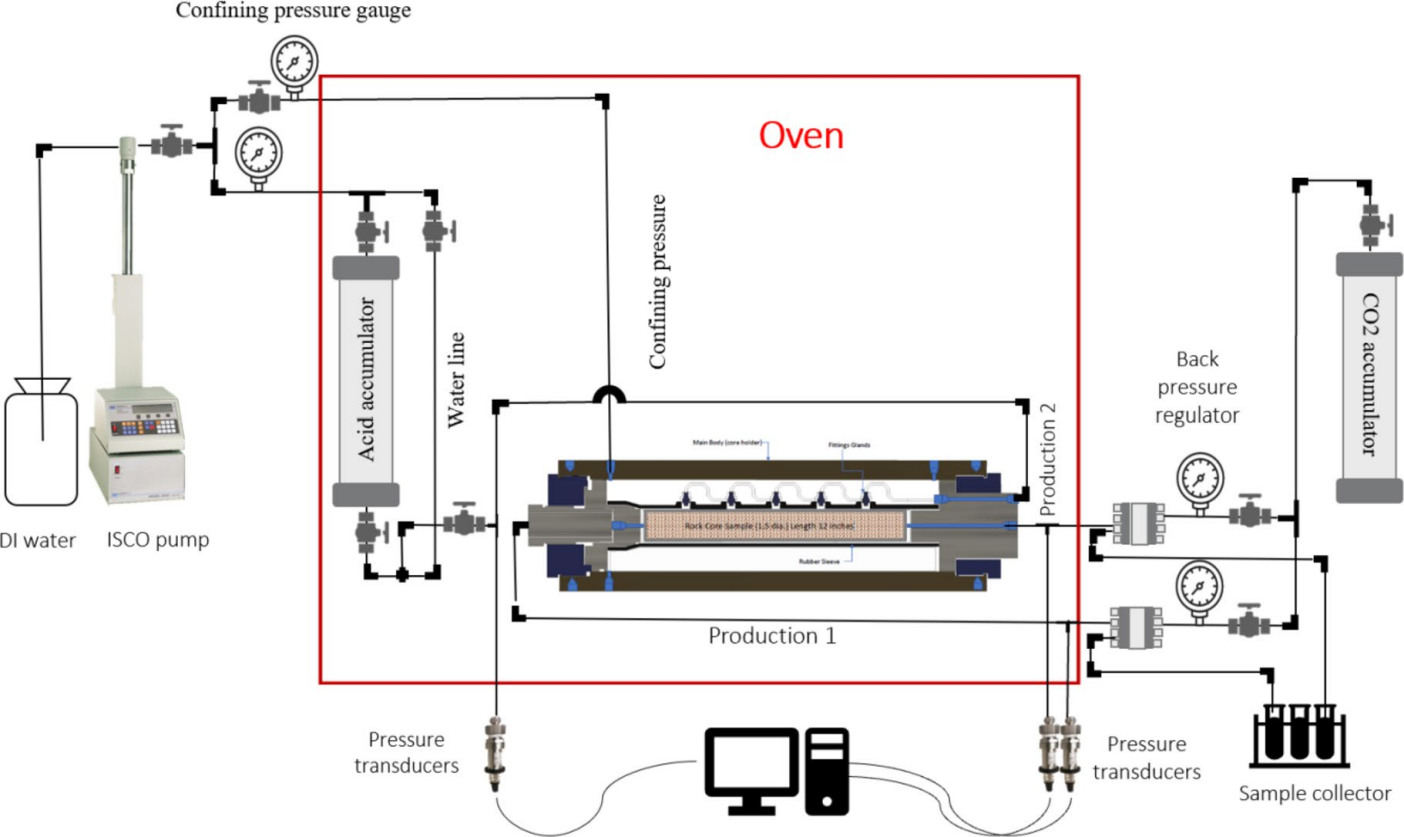
Schematic drawings of the laboratory flooding system.

**Fig. 2:**
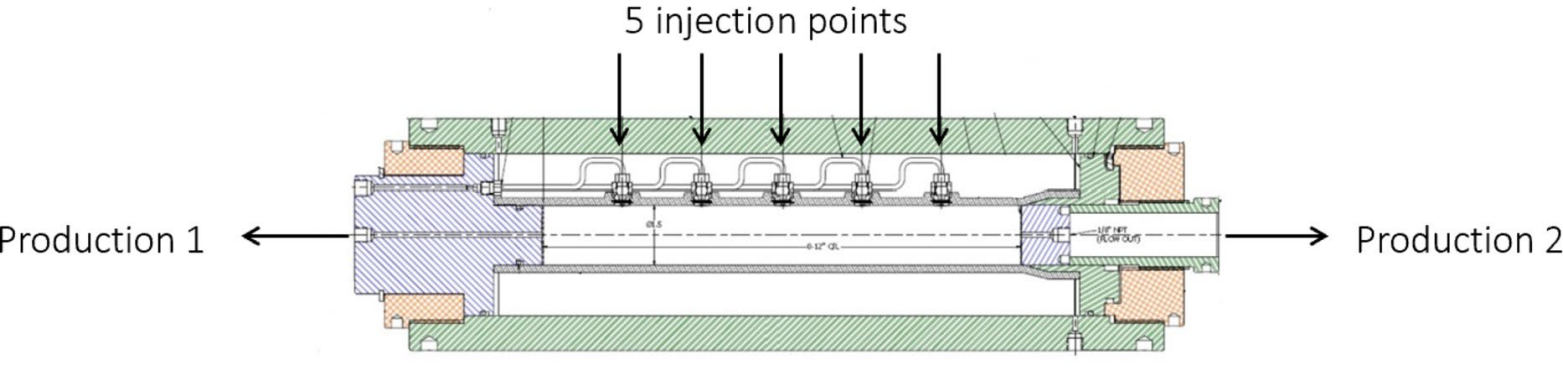
Illustration of injection points and production points.

Figure 1 shows the schematic drawings of the flooding system used for acidizing experiments. The experiments were conducted at 60 °C, 3500 psig confining, and 2000 psig back pressure. In this context, back pressure refers to the pressure used to simulate the formation pressure in an oil well. The novated flooding system can handle up to 12 inches in length and a 1.5-inch diameter core sample. The core flooding system operates under demanding conditions of 10,000 psi and 150 °C, necessitating materials like Hastelloy for the core holder and lines to withstand acidity at high temperatures. The HP/HT Coreholder, made from Hastelloy, maintains the core sample under a confining pressure of 3500 psi to regulate fluid flow. The core holder, as shown in Fig. 2, consists of five inlet points connected to accumulators holding DI water and acid system solution, while the two outlets are linked to the effluent sample collection production line. A CO2 accumulator applies 2000 psi back pressure. The 100 DX ISCO pump delivers the acid solution at a specified contact flow rate. Pressure transducers monitor pressure differentials between the inlet and outlet, while a fraction collector gathers effluent samples. These components comprise a robust system for conducting experiments under simulated reservoir conditions.

#### X-ray micro-computed tomography

Computed tomography is a non-destructive technique that uses X-rays to generate a series of 2D cross-sectional images of a rock sample. Then, a 3D model of the sample may be created by PerGeos software using these photos. The images can be analyzed using software to determine porosity, permeability, mineral composition, wormhole, and pore size distribution within the rock sample. The CT scan consists of four parts: an X-ray source system, a mechanical motion system, a data-gathering system, and an image construction system. By obtaining the typical curve, wormhole length can be objectively determined^28,42^. The X-ray source produces an X-ray that passes and attenuates through the rock, which varies based on mineral density and is then received by a detector to create a projection image. This process is repeated by rotating the rock sample 180 or 360 degrees to generate a series of projection images^43^.X-ray micro-computed tomography scan images (micro-CT) of the core samples after HCl injection were taken to capture the dissolution volume and pattern. The scan was taken through a Zeiss VersaXRM-500 X-ray micro-CT scanner with 47 μm resolution.

### Experimental procedure

The experimental procedure commenced with measuring sample porosity using a helium porosimeter (AP-608) under API RP 40. Subsequently, the samples underwent a meticulous preparation process, which involved vacuuming for three hours and saturation with di-water for 24 h, following API RP 40 guidelines for proper core handling and fluid saturation. The samples were carefully loaded into a core holder and placed inside the oven. A confining pressure of 3500 psi was then applied, and the oven was heated to 60 °C, with a waiting period of three hours to ensure uniform temperature distribution across all parts of the flooding system. Once equilibrium was reached, the inlet and outlet pressures were recorded, signifying the commencement of the core flooding process with di-water before the injection of the acid system. Upon completion of the acid injection, the system underwent a post-flush with di-water. Subsequently, the samples were offloaded and transferred to a drying oven set at 100 °C for 24 h, following the API RP 40 recommendations for post-experiment sample drying. Finally, computerized tomography was utilized to visualize the generated wormholes, adhering to the guidelines provided in ASTM E1441-11, thereby providing crucial insights into the effectiveness of the experimental procedure.

### Experimental design

A systematic approach was undertaken in the series of four experiments aimed at understanding wormhole propagation in porous media. In the first experiment, neat hydrochloric acid (HCl) was injected to establish a diversion behavior baseline. Breakthrough refers to the point at which the injected acid penetrates the outer surface of the sample and forms a continuous flow path (wormhole) between the injection point and one of the outlets. Subsequently, viscoelastic surfactants (VES) were introduced alongside HCl in the second experiment, with injection stopped after the breakthrough to analyze the immediate impact on wormhole formation. The third experiment extended this inquiry by continuing injection after the breakthrough to evaluate the continuous influence on wormhole propagation. Finally, a heterogeneous sample with varying permeability was introduced to simulate heterogeneous conditions. By systematically varying parameters and conditions across these experiments, a comprehensive understanding of wormhole propagation mechanisms and the influence of different factors on diversion behavior was gained, offering valuable insights for enhanced acid diversion strategies.

**Table 1:** Details of experiments.

Experiment No.	Core No.	Core dimensions	Inj. rate (cc/min)	Acid system	Permeability (mD)	Porosity (%)
1	IL1	1.5 in × 12 in	1	15 wt% HCl + 1% CI	16	15.77
2	IL2	1.5 in × 12 in	1	15 wt% HCl + 6 wt% VES + 10% CaCl_2_ + 1% CI	16	16.02
3	IL3	1.5 in × 12 in	1	15 wt% HCl + 6 wt% VES + 10% CaCl_2_ + 1% CI	16	16.61
4	IL4	1.5 in × 6 in 1.5 in × 6 in	1 1	15 wt% HCl + 6 wt% VES + 10% CaCl_2_ + 1% CI	10	16.47
	IL5				50	19.06

## Results

### Core flooding results

#### Neat acid system

An Indiana limestone core of diameter 1.5 in and length 12 in, with permeability around 16 mD, was used in this experiment. The acid system used in this experiment, as shown in Table 1, consists of 15 wt% HCl mixed with 1% CI. As mentioned in the experimental procedure, the experiment started with di-water injection as a pre-flush to fully saturate the sample. Injection of the acid system began, and 9 min later the breakthrough and then after 15 min at outlets 2 and 1, respectively. According to the plan, the acid injection continued for 25 min after the breakthrough in both outlet points to check the wormhole propagation. This was followed by di-water flushing the system.

**Fig. 3:**
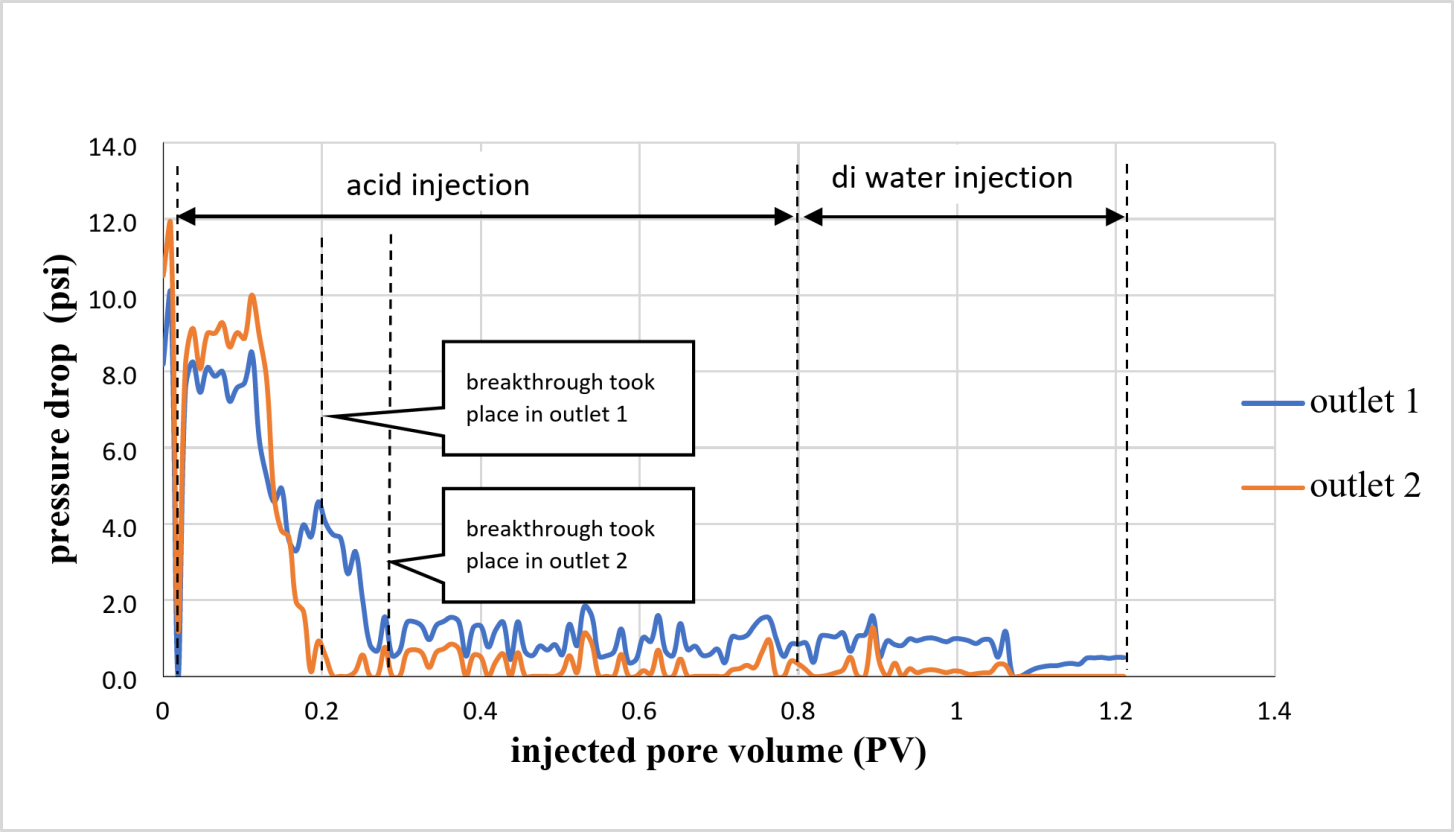
Pressure drop of a cross IL1 sample during 15wt% HCl acid injection.

Figure 3 illustrates the relationship between the pressure drop and the number of pore volumes injected. The breakthrough occurred at outlet 2 after a total of 0.19 PV of acid had been injected. At outlet point 1, a total of 0.27 PV was used before the breakthrough took place.

#### VES acid system (injection stops after breakthrough)

Indiana limestone core of diameter 1.5 in and length 12 in, with permeability around 16 mD, was used in this experiment. The acid system of this experiment, as shown in Table 1, consists of 15 wt% HCl, 10 wt% CaCl_2,_ and 6 wt% of VES mixed with 1 wt % CI. As mentioned in the experimental procedure, the experiment started with di-water injection as a pre-flush to fully saturate the sample. Then, after injecting the acid system, the breakthrough took place from outlet 2 after 5 min, and 23 min after the experiment started the breakthrough took place in outlet 1. According to the plan, the acid injection was stopped after the breakthrough occurred in outlet 1. Then, the system was flushed with di-water.

**Fig. 4:**
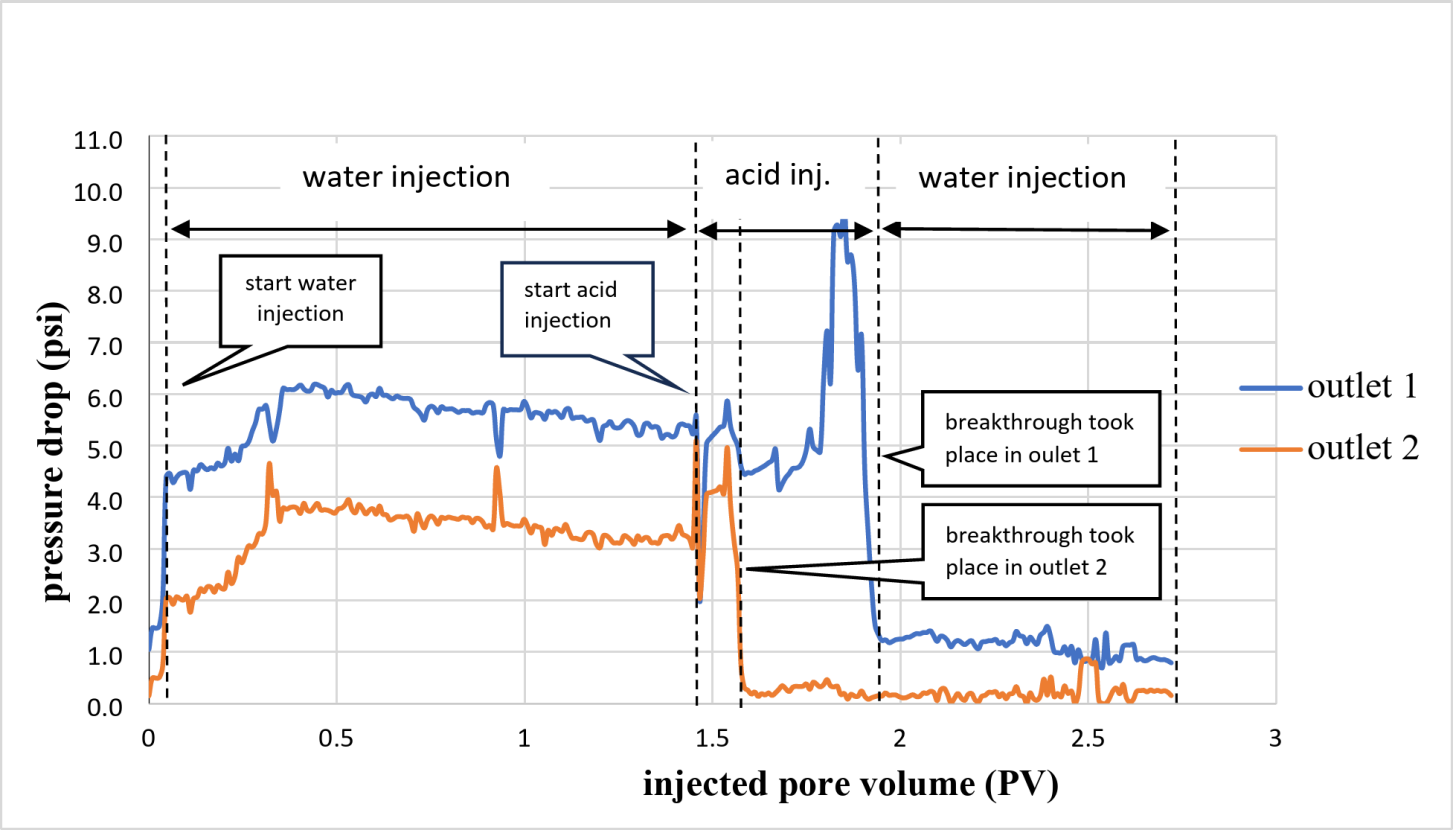
Pressure drop of a cross IL2 sample during the 15 wt% HCl acid injection mixed with 6 wt% VES.

As shown in Fig. 4, the breakthrough occurred on the high permeability side (outlet 2), with a lower pressure difference than the other part. After that, the flow stopped in outlet 2 due to the increased viscosity of the spent acid, which led to temporary blockage, and the acid diverted to the lower permeability side (outlet 1).

Figure 4 shows the pressure drop versus the number of pore volumes injected. The breakthrough occurred in outlet point 2 after pumping 1.6 pore volume (PV) of the acid. While in outlet point 1, 2 PVs were consumed until the breakthrough occurred.

#### VES acid system (injection continues after breakthrough)

Indiana limestone core, 1.5 in diameter and 12 in length, with permeability around 16 mD, was used in this experiment. The acid system used in this experiment, as shown in Table 1, consists of 15 wt% HCl mixed with 10 wt% CaCl_2_, 6 vol% VES, and 1% CI. As mentioned in the experimental procedure, the experiment started with di-water injection as a pre-flush to fully saturate the sample. Then, after injecting the acid system, the breakthrough took place from outlet 2 after 10 min, and 115 min after the acid injection started, the breakthrough took place in outlet 1. Then, following the plan, the injection of acid was continued even after the breakthrough took place in both of the outlet points in this experiment. Finally, the system was flushed with di-water.

**Fig. 5:**
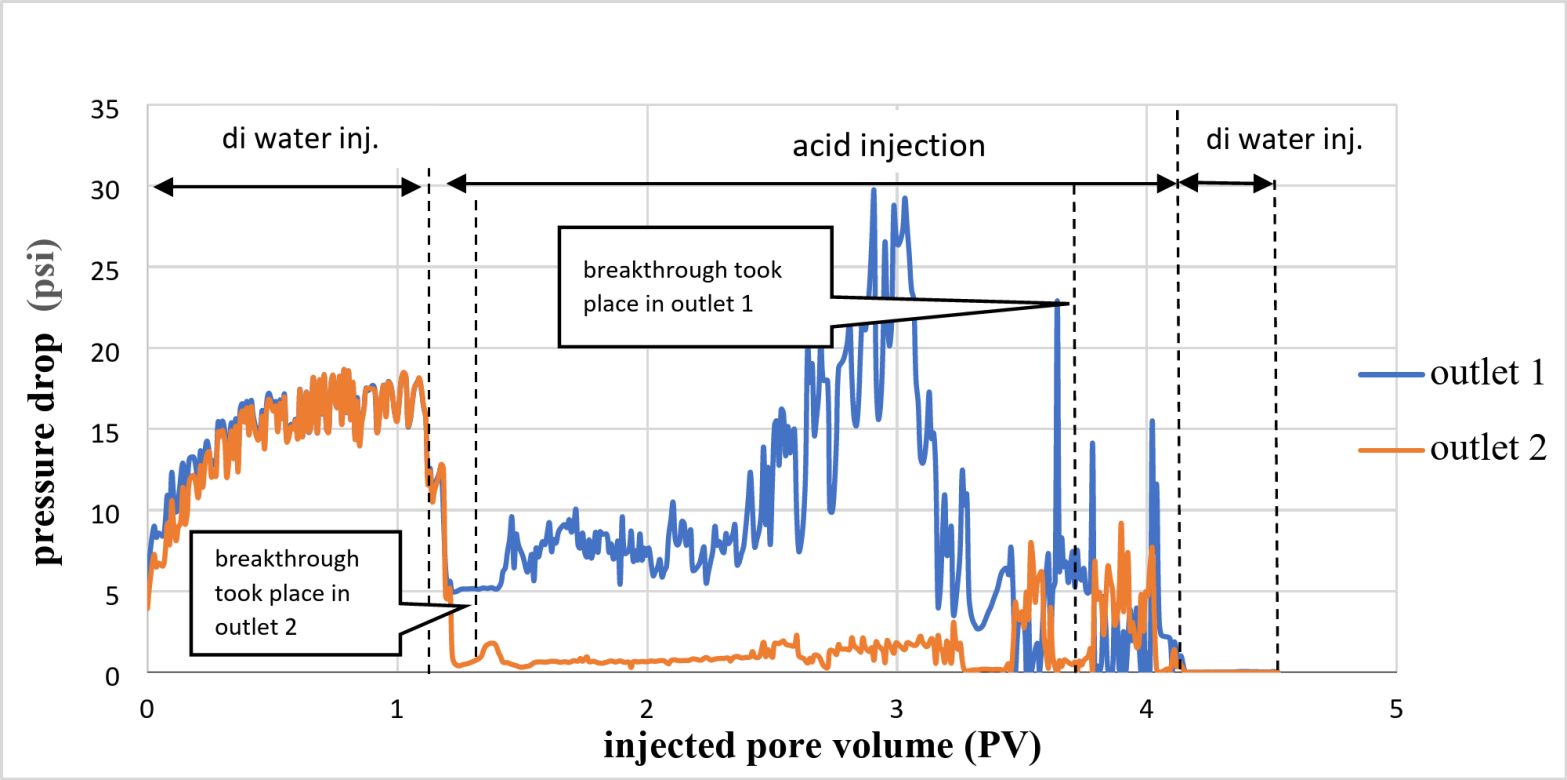
Pressure drop of a cross IL3 sample during the 15 wt% HCl acid injection mixed with 6 wt% VES.

As shown in Fig. 5, the breakthrough occurred on the high permeability side (outlet 2), with a lower pressure difference than the other part. After that, the flow stopped in outlet 2 due to the increased viscosity of the spent acid, which led to temporary blockage, and the acid diverted to the lower permeability side (outlet 1).

Figure 5 shows the pressure drop versus the number of pore volumes injected. The breakthrough occurred in outlet point 2 after pumping 0.22 PV of the acid. In outlet point 1, a total of 2.4 PV was consumed before the breakthrough occurred. After the breakthrough in both outlet points, the flow alternated between the first and second outlet points due to increased viscosity, which caused temporary flow blockage, leading the flow to switch to the other direction.

#### VES acid system (heterogenous permeability)

In this experiment, two Indiana limestone samples were used simultaneously. The diameter of the samples was 1.5 in, and the length was 6 in. The permeabilities of the cores were different; the tight one had a permeability equal to 10 mD, while the one representing a high permeability zone had 50 mD.

The acid system used in this experiment, as shown in Table 1, consists of 15 wt% HCl mixed with 10 wt% CaCl_2_, 6 wt% VES, and 1 wt% CI. As mentioned in the experimental procedure, the experiment started with di-water injection as a pre-flush to fully saturate the sample. Then, followed by injecting the acid system, the breakthrough took place in the high permeability core (outlet 2) after 26 min, and then 31 min after the acid injection began (a 5 min gap time), the breakthrough took place in the low permeability core (outlet 1). The injection of acid continued after the breakthrough took place in both outlet points in this experiment. Finally, the system was flushed with di-water.

**Fig. 6:**
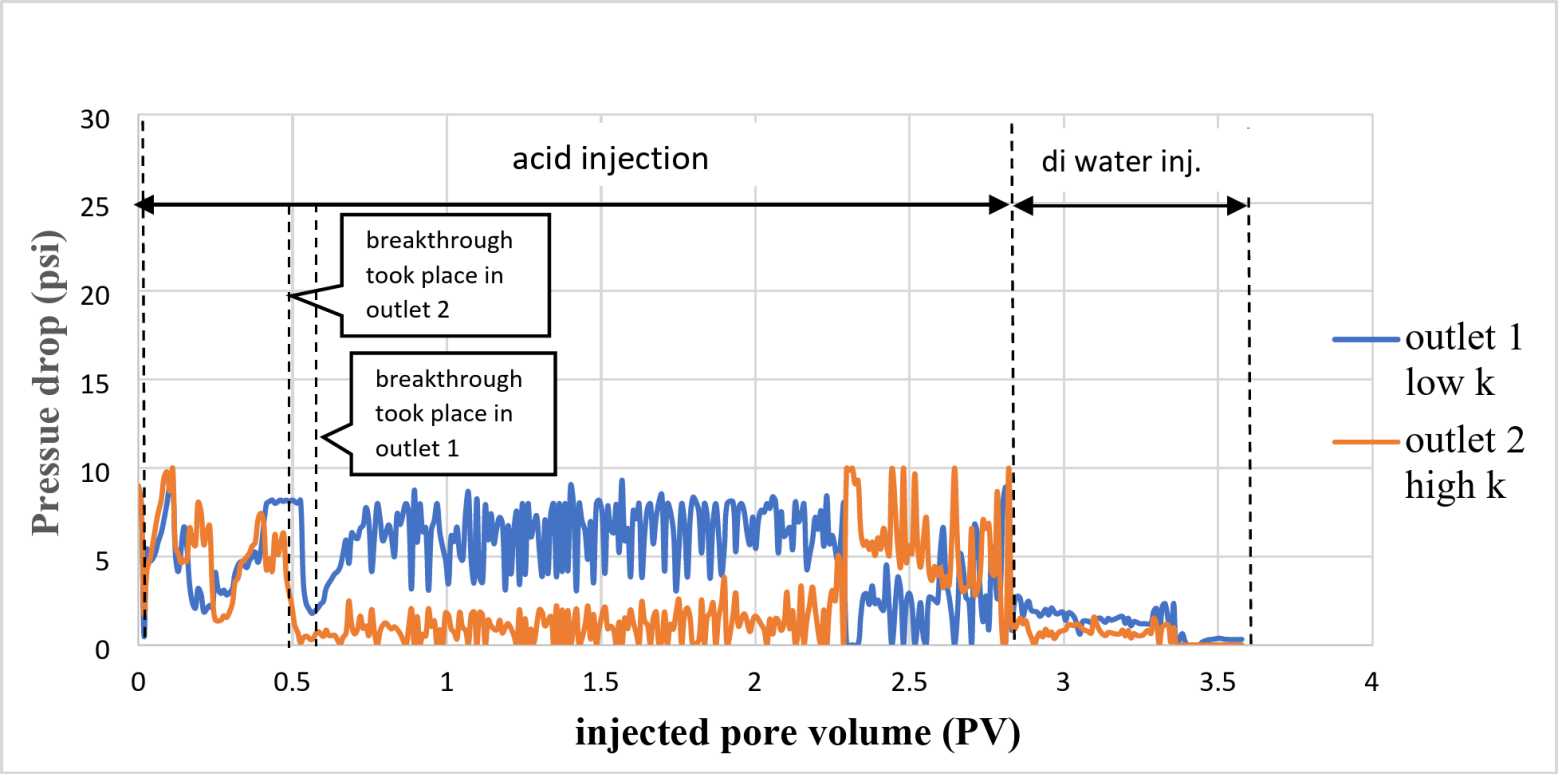
Pressure drop of a cross IL4 and IL5 sample during the 15 wt% HCl acid injection mixed with 6 wt% VES.

As shown in Fig. 6, after the breakthrough occurred in both core samples, the flow from the high permeability core stopped. Flow continued from the low permeability core, the wormhole widened without diversion to the other side until 60 min; later, the flow alternated between outlet points 1 and 2.

Figure 6 shows the pressure drop versus the number of pore volumes injected. The breakthrough occurred in outlet point 2 after pumping 0.5 PV of the acid. While in outlet point one, 0.77 PVs were consumed until the breakthrough occurred. After the breakthrough in both outlet points, the flow alternated between the first and second outlet points due to increased viscosity. This is caused by temporary flow blockage, leading the flow to switch to another direction.

### Computerized tomography (CT) scan results

A computerized tomography (CT) scan was used after the core flooding experiments to visualize the wormholes created and to estimate their volume. Table 2 summarizes the parameters of the wormholes generated in the experiments shown here; CT scan results determine the values.

**Table 2:** Main parameters of generated wormholes according to CT scan results.

Sample No	Wormhole volume fraction	Sample length (mm)	BV (mm^3^)	Wormhole volume (mm^3^)	Wormhole diameter (mm)
IL1	0.018	305.21	340.70	6.27	0.078
IL2	0.030	305.30	340.80	10.38	0.100
IL3	0.034	305.24	340.73	11.65	0.106
IL4	0.025	152.21	179.97	4.63	0.095
IL5	0.024	152.38	174.64	4.19	0.091

**Fig. 7:**
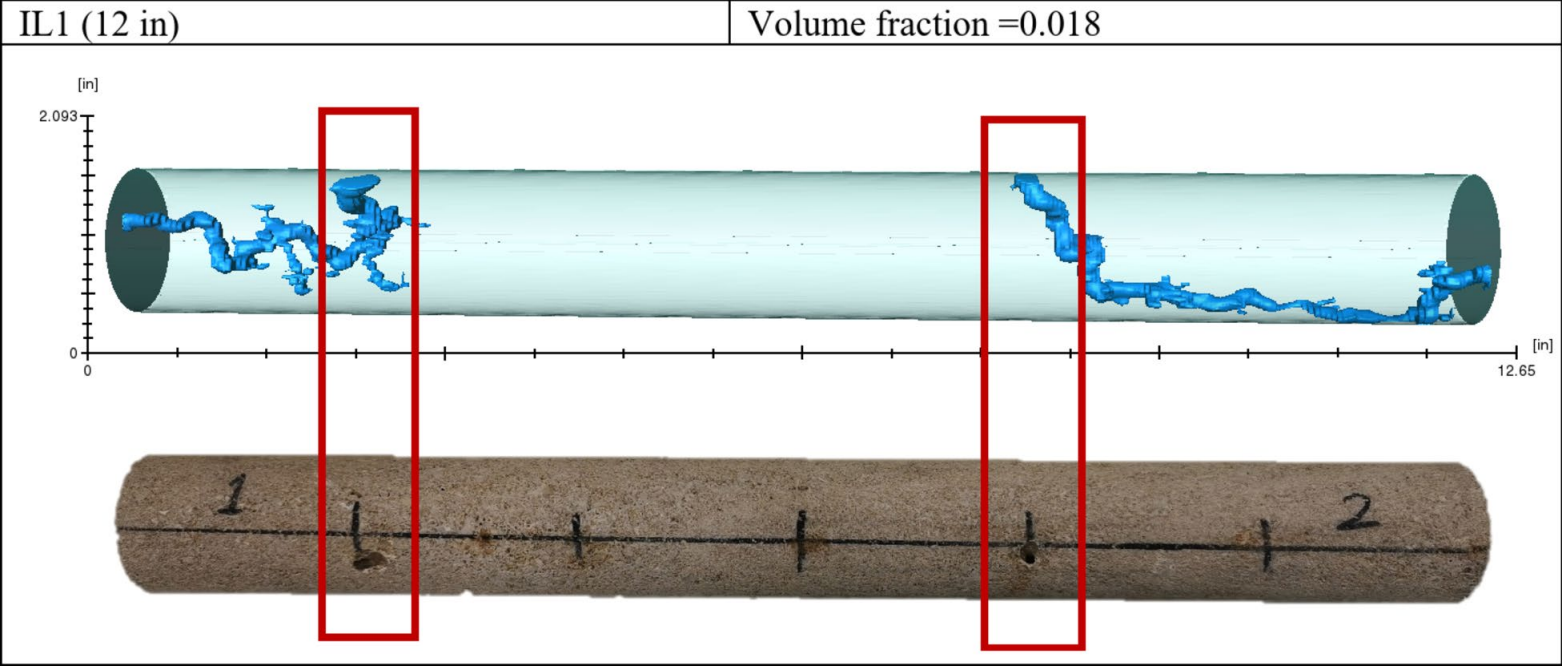
Wormholes generated during Experiment 1.

In the first experiment, hydrochloric acid was used, and the wormhole was generated on both sides with a short time gap between the first and second breakthrough. As shown in Fig. 7, there was no diversion in the wormhole, even with continued acid injection after the breakthrough.

**Fig. 8:**
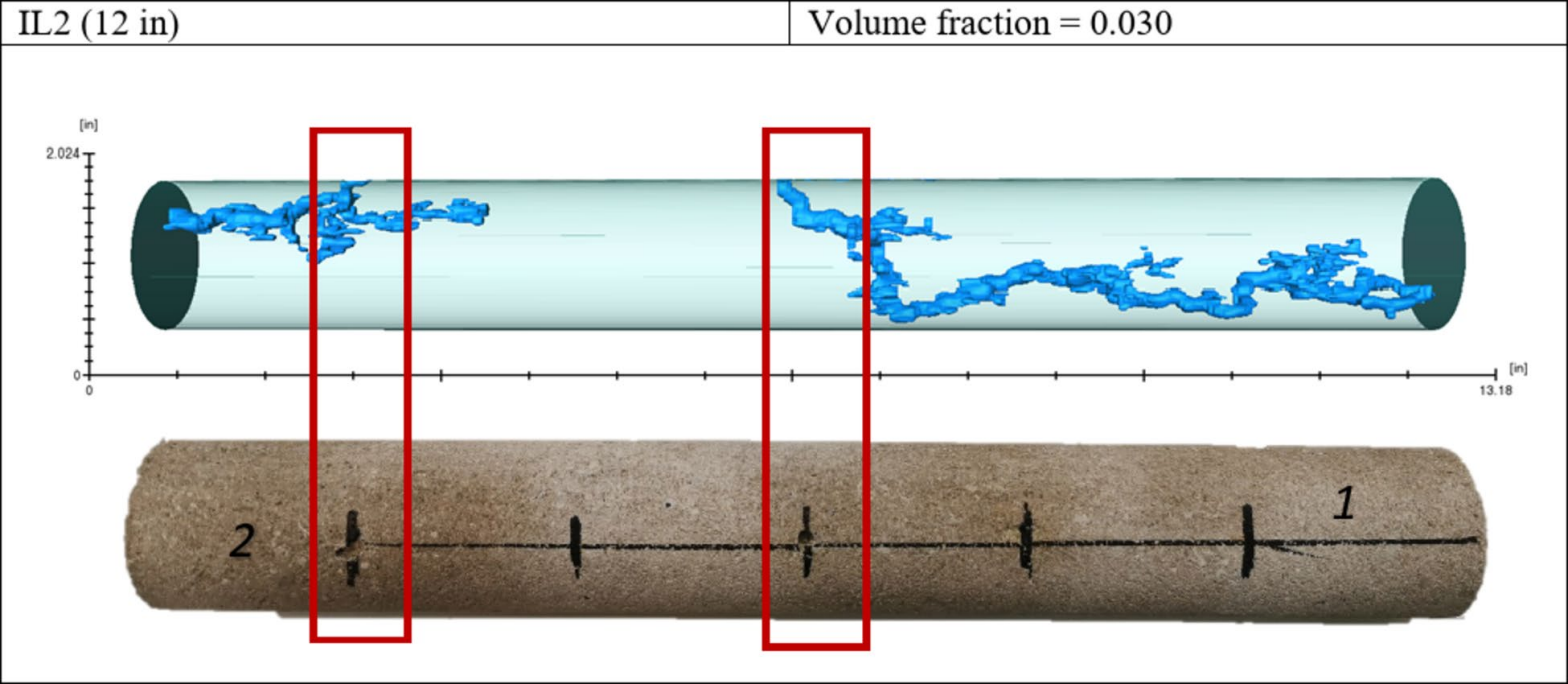
Wormholes generated during Experiment 2.

Figure 8 shows the CT scan result of Experiment 2. In that case, the wormhole was generated on the high permeability side and then diverted to the lower permeability side. The wormhole did not divert to the opposite side because acid injection stopped after the breakthrough occurred on both sides.

**Fig. 9:**
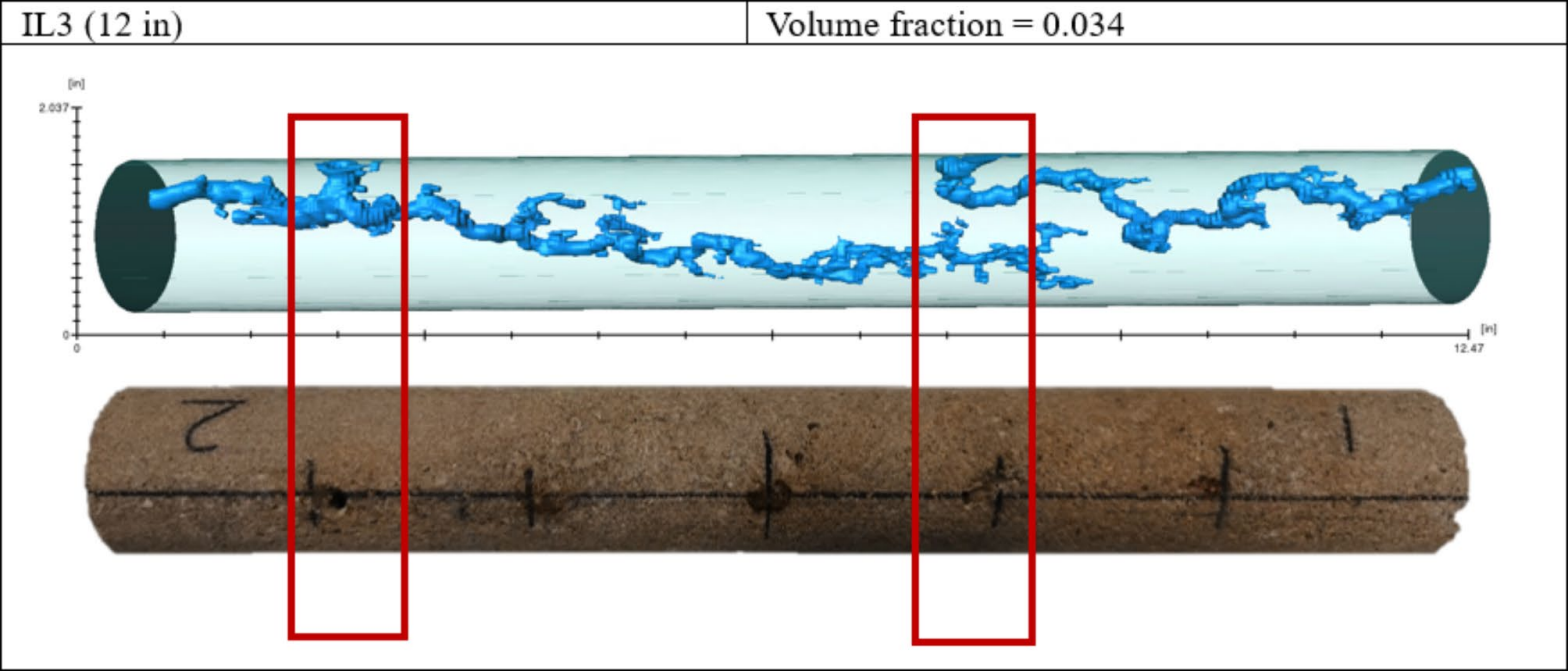
Wormholes generated during Experiment 3.

In the third experiment, hydrochloric acid with viscoelastic surfactant was used; the wormhole was first generated on the high permeability side from the closest spot from the edge, and then the acid diverted to the other side, as shown in Fig. 9. As indicated in Table 2, the wormhole volumes generated in Experiments 2 and 3 are close to each other 11.6 and 10.3, respectively. However, the wormhole length was more significant in experiment 3 due to the effect of acid diversion, which created a long wormhole with less acid volume consumption.

**Fig. 10:**
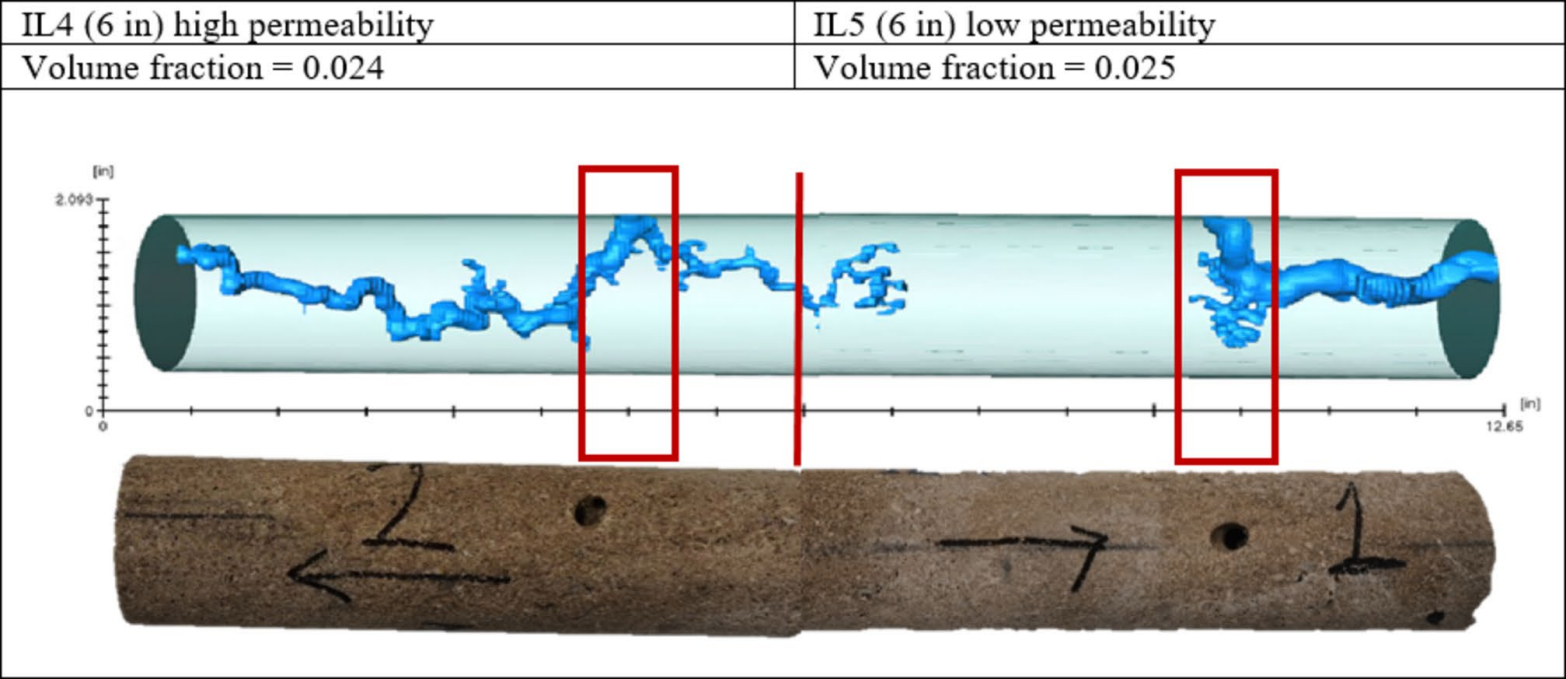
Wormholes generated during Experiment 4.

In Experiment 4, hydrochloric acid with viscoelastic surfactant was used; the wormhole was created in the high permeability side (point 2) at first, and then the flow slowed down after the breakthrough in outlet 2, and the flow increased from outlet 1 and after 4-minutes the breakthrough occurred in point 1. As shown in Fig. 10, the wormhole generated in the low permeability core was wider and had no diversion from the opposite direction; on the contrary, the wormhole generated in the high permeability core was thinner and generated in both directions on the initiated spot.

## Discussion

The introduction of the novel multi-point injection system offers a significant advantage in evaluating the diversion efficiency of certain chemicals during matrix acidizing in horizontal wells. This innovative design aims to replicate the perforation distribution in horizontal wells more accurately, thereby improving acid distribution and diversion efficiency by incorporating five injection points and two outlet lines. This allows researchers to systematically assess the performance of chemical diverters, such as the viscoelastic surfactants (VES) utilized in our study, in directing acid flow across heterogeneous carbonate formations.

The flooding outcomes from the four experiments utilizing the novel multi-point injection technique provide significant observations regarding acid distribution and diversion performance during matrix acidizing in horizontal wells. Experiment 1 (IL1), which used hydrochloric acid (HCl) only, showed limited diversion, resulting in the formation of wormholes on both sides of the sample, highlighting the need for improved acid placement strategies. Experiments 2 (IL2) and 3 (IL3), which used HCl with viscoelastic surfactants (VES), showed more successful diversion compared to the first experiment, as evidenced by higher wormhole volumes and longer lengths. Experiment IL4 demonstrated the potential for improved diversion in heterogeneous reservoir conditions using two samples with different permeabilities.

In experiments 3 and 4 (HCL with VES), wormhole propagation occurred on both sides due to the flow fluctuation. This phenomenon was attributed to the temporary blockage caused by viscosity change. When the acid flows on one side, the reaction of the acid with calcite induced a structural transformation in the molecule of VES from spherical to rod-shaped, leading to wormlike micelles. The viscosity increased locally due to this transformation, which created a temporary blockage. Consequently, the flow is redirected to the previously blocked side. This process was repeated cyclically, alternating the flow on both sides. As a result, the wormhole was longer on both sides than in other experiments.

The diversion enhancement observed in flooding experiments is due to the rheological behavior of viscoelastic surfactant (VES) under the experimental conditions. We conducted rheological tests on the fluid at 60 °C and observed the viscosity generated with acid at various shear rates. The results showed shear-thinning behavior, where viscosity decreased as the shear rate increased. This behavior is crucial because the viscosity increment at a low shear rate creates the flow resistance in the high permeability zone, which, in turn, redirects the flow to the low permeability zone. The rheological tests confirmed that the VES produced a significant increase in viscosity, reaching up to 153 cP, validating the enhanced flow resistance and effective diversion caused by the VES.

The computed tomography (CT) scan results demonstrate the new multi-point injection technique’s efficacy in evaluating the diversion efficiency of specific chemicals during matrix acidizing in horizontal wells. The wormhole volumes of Samples IL2 and IL3 are much higher, which suggests that their diversion efficiency is improved, mainly when using chemical diverters such as viscoelastic surfactants (VES). The results emphasize the significance of utilizing modern CT scans to assess acid diversion schemes precisely, resulting in improved reservoir stimulation techniques and optimum hydrocarbon extraction.

Investigations of this novel approach provide insights into acid diversion behavior and wormhole propagation mechanisms. The study highlights the influence of chemical diversion on acid placement and distribution by systematically evaluating the efficiency of different acid systems, such as hydrochloric acid (HCl) alone and HCl mixed with VES. The experiments demonstrate that introducing VES leads to longer and more effectively distributed wormholes, indicating improved diversion efficiency compared to conventional methods.

## Conclusions and recommendations

This study used a new flooding system to better understand acidizing in horizontal wells and to identify how the acid behaves and diverts in heterogeneous carbonate formation using viscoelastic surfactant (VES) as a diverter solution. Computerized tomography (CT) scans were also used to visualize the wormholes generated inside the rock samples. The findings and conclusions that resulted from this research can be briefly described as follows:

- Wormhole locations initiated in each experiment differed from each other due to the heterogeneity of the formation.
- In each experiment, wormholes occurred far from each other. This suggests that the generated wormholes are competing.
- In all of the experiments conducted, only two wormholes were created out of five injection points, meaning not all the perforations in the horizontal wells can receive acid.
- Continued injection after the breakthrough, as in Experiment 3, led to a longer wormhole than in Experiment 2, where injection was stopped after the breakthrough.
- In both Experiment 1 (HCl only) and Experiment 3 (HCl + VES), injection continued after breakthrough, but the wormhole generated in Experiment 3 was longer than that in Experiment 1.
- When using only HCl, the continuation of injection after the breakthrough leads to a wider wormhole, which is opposite to the effect of using HCl with VES, which produced a longer wormhole with less acid consumption.

## Data Availability

The datasets used and/or analysed during the current study available from the corresponding author on reasonable request.
